# Extraction of Astaxanthin and Lutein from Microalga *Haematococcus pluvialis* in the Red Phase Using CO_2_ Supercritical Fluid Extraction Technology with Ethanol as Co-Solvent

**DOI:** 10.3390/md16110432

**Published:** 2018-11-03

**Authors:** Antonio Molino, Sanjeet Mehariya, Angela Iovine, Vincenzo Larocca, Giuseppe Di Sanzo, Maria Martino, Patrizia Casella, Simeone Chianese, Dino Musmarra

**Affiliations:** 1 ENEA, Italian National Agency for New Technologies, Energy and Sustainable Economic Development. Department of Sustainability—CR Portici. P. Enrico Fermi, 1, 80055 Portici (NA), Italy; antonio.molino@enea.it (A.M.); sanjeet.mehariya@unicampania.it (S.M.); angela.iovine@unicampania.it (A.I.); patrizia.casella@enea.it (P.C.); 2 Department of Engineering, University of Campania “Luigi Vanvitelli”, Real Casa dell’Annunziata, Via Roma 29, 81031 Aversa (CE), Italy; simeone.chianese@unicampania.it; 3 ENEA, Italian National Agency for New Technologies, Energy and Sustainable Economic Development. Department of Sustainability—CR Trisaia. SS Jonica 106, km 419+500, 7026 Rotondella (MT), Italy; vincenzo.larocca@enea.it (V.L.); giuseppe.disanzo@enea.it (G.D.S.); maria.martino@enea.it (M.M.)

**Keywords:** CO_2_ supercritical fluid extraction, *Haematococcus pluvialis*, lutein, astaxanthin, ethanol, co-solvent, antioxidants, purity, recovery, extraction

## Abstract

Astaxanthin and lutein, antioxidants used in nutraceutics and cosmetics, can be extracted from several microalgal species. In this work, investigations on astaxanthin and lutein extraction from *Haematococcus pluvialis* (*H. pluvialis*) in the red phase were carried out by means of the supercritical fluid extraction (SFE) technique, in which CO_2_ supercritical fluid was used as the extracting solvent with ethanol as the co-solvent. The experimental activity was performed using a bench-scale reactor in semi-batch configuration with varying extraction times (20, 40, 60, and 80 min), temperatures (50, 65, and 80 °C) and pressures (100, 400, and 550 bar). Moreover, the performance of CO_2_ SFE with ethanol was compared to that without ethanol. The results show that the highest astaxanthin and lutein recoveries were found at 65 °C and 550 bar, with ~18.5 mg/g dry weight (~92%) astaxanthin and ~7.15 mg/g dry weight (~93%) lutein. The highest astaxanthin purity and the highest lutein purity were found at 80 °C and 400 bar, and at 65 °C and 550 bar, respectively.

## 1. Introduction

Currently, the interest in microalgae is on the rise, as the knowledge of their benefits in several aspects of human life increases. Astaxanthin and lutein, high-value molecules for many industrial sectors, e.g., pharmaceuticals, nutraceuticals, additive/functional foods, natural medicine, and cosmetics, can be extracted from microalgal species [1,2]. Microalgae can live both in freshwater and seawater environments, using their photosynthesis activity to convert atmospheric carbon dioxide into oxygen and sugars in the presence of sunlight [3]. During their life cycle, they can be used for carbon dioxide sequestration; in fact, for each kilogram of dry biomass, about 1.8–2 kg of CO_2_ can be captured.

Astaxanthin and lutein are two very valuable carotenoids on the market. Their properties make them useful as food additives. Thanks to its antioxidant and antiaging properties, astaxanthin is also used in the cosmetic sector [4], while lutein is also used as a dietary supplement for its beneficial effects on eye health [5].

A number of factors, such as the type of molecule, the end use of the extract (e.g., pharmaceutical, cosmetics, and food), and its thermolability, affect the selection of a proper extraction process [6]. Squeezing, maceration, infusion, percolation, steam distillation, and solvent extraction are traditional techniques for extracting principal components from vegetables. The main drawbacks of these techniques are the thermal degradation of molecules, due to the high temperatures of extraction, and the presence of solvent residues in the extracts, which can compromise their end use. In order to solve these issues and, at the same time, minimize energy costs and environmental impact, innovative extraction methods, such as ultrasonic extraction, microwave extraction, accelerated solvent extraction, and extraction with supercritical fluid, were proposed by the research community in the past few decades [7,8,9,10,11,12]. 

Extraction with supercritical fluid (SFE) is an advanced technology with great potential for the extraction of molecules that require high standards in terms of yield without any traces of solvents, which is especially important when the extracts are intended for nutraceutics [13,14,15,16,17]. Extraction with supercritical fluid using CO_2_ in supercritical condition as the extracting solvent (CO_2_-SFE) is generally applied [18] as an alternative to traditional extraction technologies [19,20,21,22,23], to extract natural compounds. While the use of CO_2_ is the most common approach, many substances can be used in supercritical conditions (hexane, methanol, pentane, butane, nitrous oxide, sulfur hexafluoride, and fluorinated hydrocarbons). Thanks to its critical conditions (*P_c_* = 7.38 MPa, *T_c_* = 31.1 °C), particularly in terms of low critical temperature, it is possible to extract thermally unstable substances with CO_2_-SFE while reducing thermal degradation effects [24]. Moreover, CO_2_ is non-flammable and less toxic than conventional solvents. After extraction, CO_2_ can easily be recovered for successive cyclic extractions, since it is in a gaseous state at room temperature and atmospheric pressure [25,26]. Unlike traditional solvents with larger molecules, carbon dioxide (CO_2_) in supercritical conditions spreads faster through cellular walls thanks to its high permeability and diffusivity. Because of these advantages, CO_2_-SFE appears to be the preferable extraction process when high-quality standards are required to extract high-value compounds [27,28,29,30,31].

The main drawback of this technique is the chemical behavior of CO_2_, similar to lipophilic solvents, i.e., better able to extract non-polar molecules. In order to overcome this obstacle, substances with high polarity such as water, methanol, or ethanol and other polar compounds (called co-solvents) are often used [32,33]. Their choice depends on aspects like polarity, toxicity, and environmental impact [34,35,36]. Among co-solvents, ethanol, a generally recognized as safe (GRAS) solvent, according to the Food and Drug Administration classification [37], is often used. Actually, ethanol is normally employed in the pharmaceutical and food industry, since 50 mg of residual ethanol per day is acceptable for human health.

In the last twenty years, CO_2_-SFE was used to extract bio-products from over 300 vegetables [21,23], as highlighted in the many of papers and patents reported in the literature [38,39,40]. Currently, CO_2_-SFE is also used for the extraction of several active principal components from algae that can be used in the food industry [41,42,43], such as high-value lipids [44]. 

*Haematococcus pluvialis* is a microalgal species that can grow both in freshwater and in aquatic environments with considerable concentrations of NaCl [45,46,47], and it is characterized by its ability to accumulate significant concentrations of astaxanthin and lutein. Several experiments focusing on the extraction of astaxanthin from *H. pluvialis* via CO_2_-SFE were carried out without [48,49,50,51] and with ethanol as a co-solvent [50,52,53]. For example, Kwan and co-workers [51] developed a method of selectively purifying astaxanthin and other by-products (triacylglycerides) from H. pluvialis using supercritical CO_2_. Cheng et al. [50] investigated the low-pressure supercritical CO_2_ extraction of astaxanthin from *H. pluvialis* using a microfluidic reactor with and without co-solvent (ethanol and olive oil), observing the increase in astaxanthin recovery and the decrease in extraction time in the presence of a co-solvent (astaxanthin recovery = 92%, at 55 °C, 8 MPa, and 15 min). Lutein extraction via CO_2_-SFE in the presence of co-solvents from other microalgal species, such as *Chlorella* [54,55] and *Scenedesmus* [56], was also investigated. CO_2_-SFE extraction from microalgae is the starting point for the production of natural compounds with a sustainable approach and a low environmental impact.

In this paper, astaxanthin and lutein extraction from *H. pluvialis* in the red phase (HPR) was investigated using CO_2_-SFE technology with ethanol as the co-solvent. The effects of temperature (50–80 °C) and pressure (100–550 bar) on recovery and purity over extraction time (20–80 min), i.e., four extraction cycles of 20 min each, were tested by keeping flow rates of carbon dioxide and ethanol constant at 3.62 g/min and 1 mL/min, respectively, using a bench-scale experimental apparatus. Moreover, at the same operative conditions, a comparison between the experimental findings from CO_2_-SFE with ethanol and the experimental findings from CO_2_-SFE without ethanol was carried out. Experimental findings from CO_2_-SFE without ethanol were published in a previous study by Di Sanzo et al [57].

## 2. Results and Discussion

### 2.1. Extraction Yield 

Total extraction yields for each operative condition are summarized in Table 1, in which the total yields extracted without co-solvent are also included. The results are expressed as mg of extract per gram of dry weight of HPR, and values were obtained at the end of the extraction procedure. In the presence of a co-solvent, the total extraction yield ranged from 207.67 mg/g (*T* = 80 °C, *P* = 100 bar) to 292.70 mg/g (*T* = 65 °C, *P* = 400 bar); without co-solvent, the total extraction yield varied in the range of 0.1 mg/g (*T* = 50 °C, *P* = 100 bar) to 277.1 mg/g (*T* = 65 °C, *P* = 400 bar). As shown, with and without co-solvent, the highest total extraction yield was found at the same operative conditions (*T* = 65 °C, *P* = 400 bar); however, CO_2_-SFE extraction with a co-solvent increased the highest total extraction yield from 277.1 mg/g to 292.7 mg/g.

Data in Table 1 show that the total extraction yield increases as temperature and pressure increase, until a maximum is reached, before a subsequent decrease. This result is due to counteracting phenomena: increasing the pressure increases the CO_2_ density (the solvation power of the fluids [56]), but a pressure too high may obstruct the diffusion of supercritical fluid into the matrix; increasing the temperature decreases the viscosity of the solvent improving the mass transfer, but a temperature too high may degrade the extracted compounds [48,58,59]. 

### 2.2. Astaxanthin Recovery

**Figure 1:**
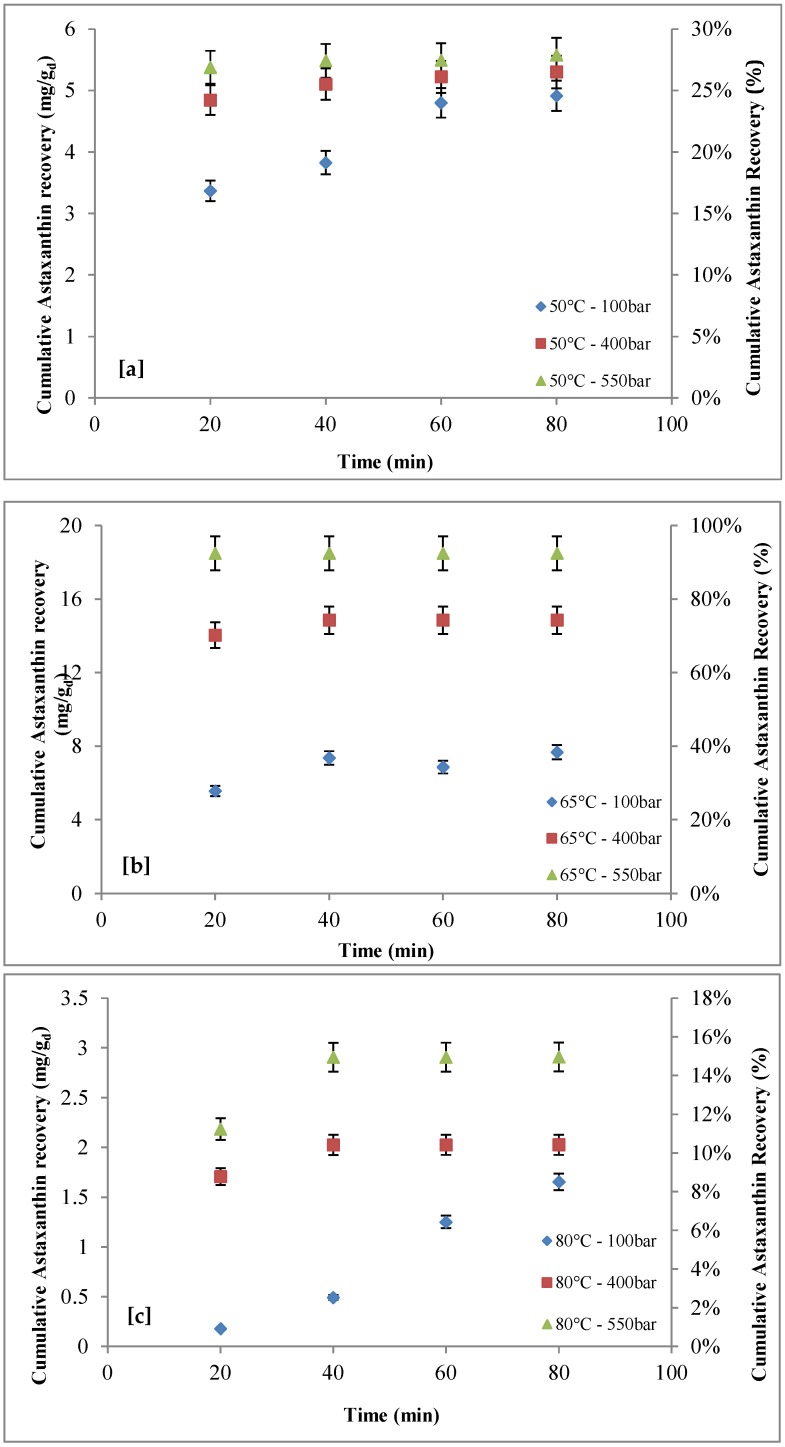
Effect of pressure (100–550 bar) on astaxanthin recovery as function of extraction time: (**a**) *T* = 50 °C; (**b**) *T* = 65 °C; (**c**) *T* = 80 °C.

**Figure 2:**
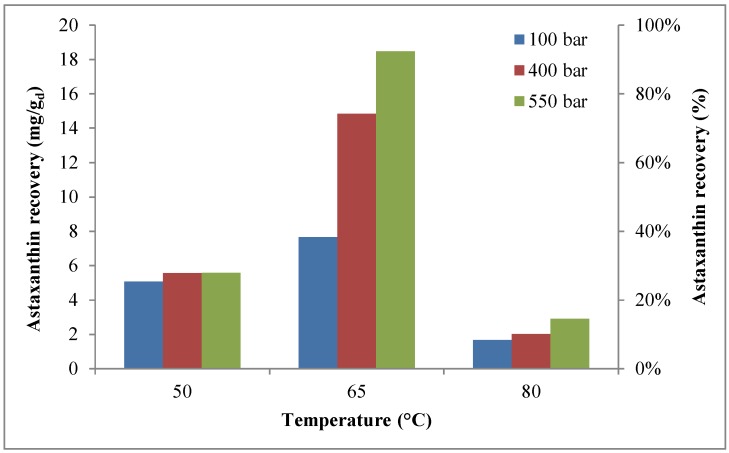
Effects of temperature (50–80 °C) and pressure (100–550 bar) on cumulative astaxanthin recovery (extraction time = 80 min).

The effects of pressure (100–550 bar), temperature (50–80 °C), and extraction time (extraction cycle number; 20–80 min) on the cumulative recovery of astaxanthin are reported in Figure 1 and Figure 2. Figure 1a–c shows that, while keeping extraction temperature constant, astaxanthin recovery increased with extraction time and pressure.

At 50 °C (Figure 1a), with an extraction time of 20 min (first extraction cycle), increasing pressure from 100 to 550 bar resulted in astaxanthin recovery growing from ~3.4 mg/g_d_ (~17%) to ~5.4 mg/g_d_ (~27%). With an extraction time of 80 min (four extraction cycles), astaxanthin extraction yield increased from ~4.9 mg/g_d_ (~25%) to ~5.6 mg/g_d_ (~28%). Increasing extraction time from 20 min to 80 min, at 100 bar, astaxanthin recovery increased from ~3.4 mg/g_d_ to ~4.9 mg/g_d_; at 400 bar, astaxanthin recovery slightly increased from ~4.8 mg/g_d_ to ~5.3 mg/g_d_; at 550 bar, astaxanthin recovery slightly increased from ~5.4 mg/g_d_ to ~5.6 mg/g_d_. At 65°C (Figure 1b) and at 80 °C (Figure 1c), for the first extraction cycle, astaxanthin recovery reached a maximum at ~18.4 mg/g_d_ (~92%) at 65 °C and 550 bar, while this value was only ~0.2 mg/g_d_ (~1%) at 80 °C and 100 bar. However, it is worth highlighting that, at 65 °C and 550 bar, astaxanthin recovery was relatively unaffected by the extraction time.

For each operative condition, the astaxanthin yield at the end of a cycle of extraction is shown in Figure 2. This figure clearly shows that, whatever the pressure, astaxanthin recovery increased with extraction temperature, reaching a maximum at 65 °C, corresponding to ~18.5 mg/g_d_ (~93%), before decreasing, probably due to thermal degradation [60,61,62]. Results show that the maximum astaxanthin recovery was obtained at the highest pressure (550 bar) and at an intermediate temperature (65 °C). It is worth pointing out that, under the best extraction conditions, a negligible influence of extraction time was basically observed, as, at 20 min, an astaxanthin extraction yield of about 18.4 mg/g_d_ was achieved, with respect to an astaxanthin extraction yield of about 18.5 mg/g_d_ reached at 80 min.

Pan et al. [63] investigated the effects of *H. pluvialis* loading, CO_2_ flow rate, time, pressure, and temperature of extraction, as well as ethanol loading, on astaxanthin extraction, observing a yield of about 71% at the following operating conditions: temperature = 50 °C, pressure = 310 bar, CO_2_ flow rate = 6 L/min, time = 160 min, and *H. pluvialis* loading = 6.5 g. Machmudah et al. [53] reported an extraction yield of about 80% at a pressure of 400 bar and a temperature of 343 K, with a CO_2_ flow rate of 3 mL/min and an ethanol concentration of 5%; a comparable finding was found by the same research group at a pressure of 550 bar and a temperature of 343 K, with CO_2_ flow rate of 3 mL/min without ethanol. It is worth highlighting that, in this study, an astaxanthin yield of about 93% at 550 bar and 65 °C, with a CO_2_ flow rate of 3.62 g/min and an ethanol flow rate of 1 mL/min was observed.

The comparison between cumulative recovery of astaxanthin with and without co-solvent [57] is reported in Table 2. As shown, the maximum recovery of astaxanthin (~94%) was found without co-solvent at 550 bar and 50 °C. However, a comparable result was achieved with co-solvent (~92%) at 550 bar and 65 °C.

The purity of astaxanthin for each stage of extraction, under different operative conditions, with and without co-solvent is reported in Table 3. With co-solvent, the highest purity (~18%) was achieved with an extraction time of 40 min at 80 °C and 400 bar. Comparable findings were observed by Reyes et al. [64], who investigated the effect of ethanol content in the CO_2_ flow on astaxanthin extraction from *H. pluvialis* by CO_2_-SFE, observing a maximum purity of about 23% at 70 °C and 275 bar with an ethanol content in the CO_2_ flow of 13% (*w*/*w*). Without co-solvent, the highest purity (~34%) was found with an extraction time of 80 min at the same pressure and temperature. 

As shown in Table 3, an effective improvement of the extraction of astaxanthin in the presence of co-solvent was not observed, despite CO_2_-SFE with ethanol showing a valuable level of astaxanthin recovery. Moreover, a reduction in purity was found; this result may be explained by considering that, in the presence of ethanol, the total extraction yield increased, reducing the purity of astaxanthin. 

### 2.3. Lutein Recovery

**Figure 3:**
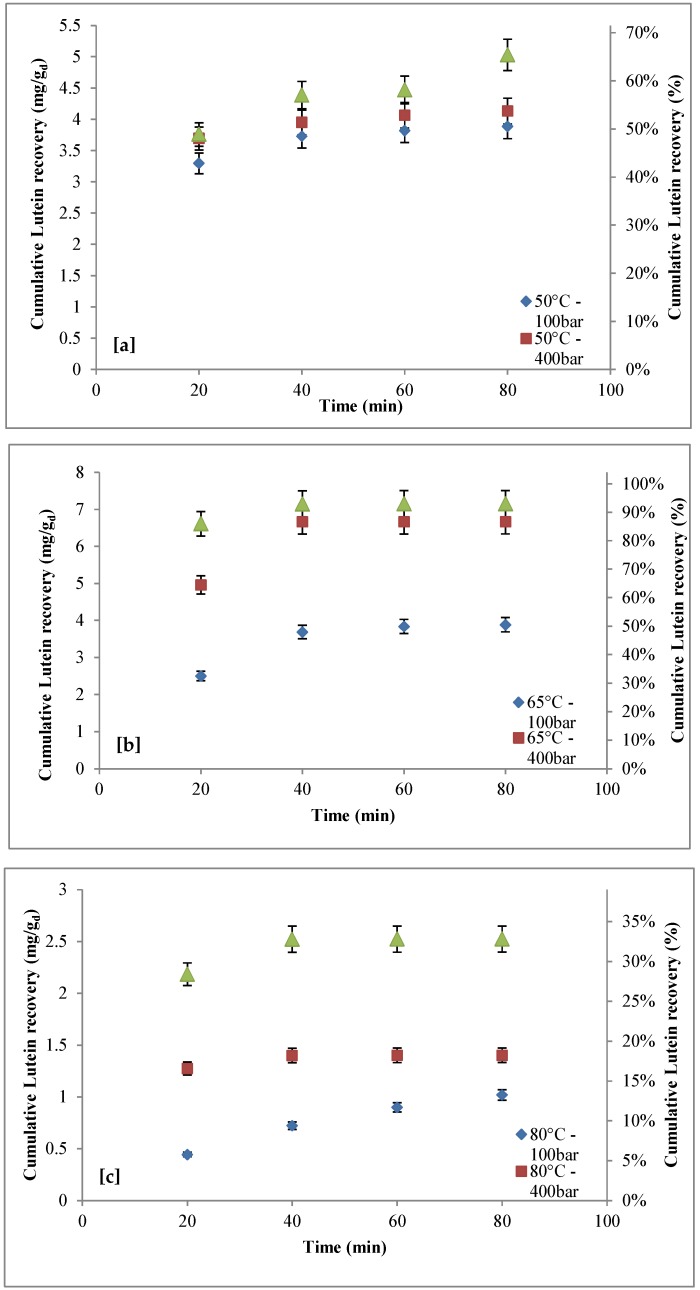
Effect of pressure (100–550 bar) on lutein recovery as function of the extraction time: (**a**) *T* = 50 °C; (**b**) *T* = 65 °C; (**c**) *T* = 80 °C.

The effects of pressure, temperature, and extraction time (extraction cycle number) on total lutein recovery are reported in Figure 3. Figure 3a–c shows that, keeping extraction temperature constant, lutein recovery increased with extraction time and pressure.

At 50 °C (Figure 3a) with an extraction time of 20 min (first extraction cycle), increasing pressure from 100 to 550 bar resulted in lutein recovery growing from ~3.3 mg/g_d_ (~43%) to ~3.7 mg/g_d_ (~49%). With an extraction time of 80 min (four extraction cycles), lutein extraction yield increased from ~3.9 mg/g_d_ (~50%) to ~5.0 mg/g_d_ (~65%). Increasing extraction time from 20 min to 80 min, at 100 bar, lutein recovery slightly increased from ~3.3 mg/g_d_ (~43%) to ~3.9 mg/g_d_ (~50%); at 400 bar, lutein recovery slightly increased from ~3.7 mg/g_d_ (~48%) to ~4.1 mg/g_d_ (~54%); at 550 bar, lutein recovery increased from ~3.7 mg/g_d_ (~48%) to ~5.0 mg/g_d_ (~65%). At 65 °C (Figure 3b) and at 80 °C (Figure 3c), the influence of extraction time on lutein recovery was quite similar to that observed at 50 °C (Figure 3a); however, the maximum lutein recovery, close to 7.15 mg/g_d_ (~93%) was reached in the second extraction cycle at 65 °C and 550 bar, before decreasing to ~0.4 mg/g_d_ (~6%) at 80 °C and 100 bar. Moreover, it is worth highlighting that, at the best extraction conditions (65 °C and 550 bar), starting from 40 min, the effect of extraction time was negligible (Figure 3b); therefore, a 40 min extraction was sufficient to achieve the maximum lutein extraction yield.

**Figure 4:**
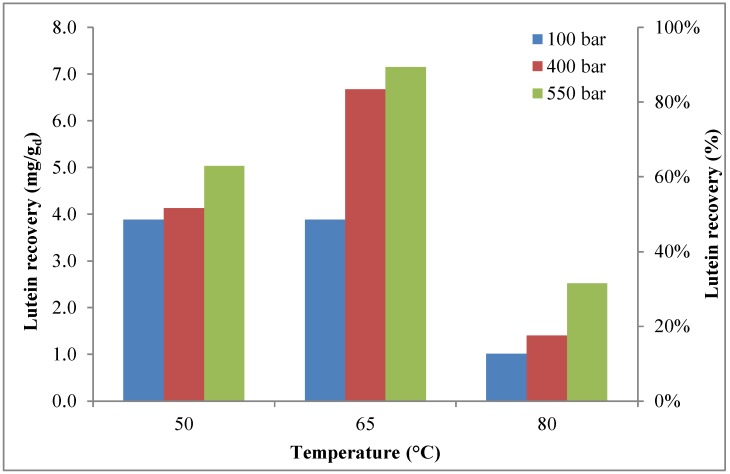
Effects of temperature (50–80 °C) and pressure (100–550 bar) on cumulative lutein recovery (extraction time = 80 min).

For each operative condition, the lutein yield at the end of a complete cycle of extraction is shown in Figure 4. This figure clearly shows that, at the pressure of 100 bar, almost the same recovery value was obtained at 50 °C and 65 °C, while, at higher extraction pressures, temperature played a crucial role, as lutein recovery increased with temperature, reaching a maximum at 65 °C before decreasing, probably due to the thermal instability of carotenoids, as reported by several authors [63,65]. At the best extraction conditions, it is possible to assume that about 93% of lutein was extracted.

To the best of the authors’ knowledge, few papers focusing on lutein extraction from *H. pluvialis* via supercritical fluid extraction were published. Nobre et al. [52] reported lutein recovery from *H. pluvialis* close to 92% at a pressure of 300 bar and a temperature of 60 °C, using CO_2_-SFE with ethanol as a co-solvent (ethanol 10%/CO_2_ 90%, (*v*/*v*)). As shown, our experimental findings are relatively in line with literature data.

The comparison between the cumulative recovery of lutein with and without co-solvent [57] is reported in Table 4. As shown, the presence of ethanol strongly improved the extraction of lutein, as the highest recovery increased from about 51% without co-solvent (*P* = 550 bar, *T* = 50 °C) to about 93% with co-solvent (*P* = 550 bar, *T* = 65 °C).

The purity of lutein for each stage of extraction, under different operative conditions, with and without co-solvent is reported in Table 5. With co-solvent, the highest purity (~16%) was achieved with an extraction time of 40 min at 65 °C and 550 bar, which are the same conditions that resulted in the highest lutein recovery, as shown in Figure 3. Without co-solvent, the highest purity (~7.5%) was found with an extraction time of 80 min at 400 bar and 50 °C. As shown, ethanol was effective for the enhancement of lutein purity, as the maximum purity increased from about 7.5% to about 16%.

### 2.4. Comparison with Literature for Recovery of Astaxanthin and Lutein Using CO_2_-SFE with Co-Solvent

The comparison between the operative conditions allowing the maximum recovery of astaxanthin and lutein defined in the present work and data available in literature is reported in Table 6. As shown, the supercritical extraction process is an effective technology for the extraction of astaxanthin and lutein, with recoveries higher than 90% for both compounds. In particular, the operative conditions identified in our study, including the type of pre-treatment, produced the highest recovery of astaxanthin and lutein. However, as shown in Figure 1 and Figure 3, in terms of the effect of extraction time on recovery, it is worth highlighting that, at the operative conditions with which the maximum recoveries were achieved (65 °C; 550 bar), the recoveries were relatively unaffected by extraction time. Therefore, the maximum recoveries of astaxanthin and lutein were found during the first extraction cycle (20 min) and the second extraction cycle (40 min), respectively, and they did not vary along with the increase in extraction time (Figure 1 and Figure 3). 

## 3. Materials and Methods

### 3.1. Samples and Chemicals

Experimental activity was carried out using lyophilized *H. pluvialis* microalgae in the red phase (HPR) provided by Micoperi Blue Growth (Ravenna, Italy). HPR has a mesh particle sieve size lower than 50 µm, with a total content of astaxanthin of 20.0 mg/g dry weight, corresponding to 2% (*w*/*w*) dry weight, which was measured using the method proposed by Li et al. [67]. Since no standard method to measure lutein content in HPR is reported in the literature, lutein content was measured using the same method proposed by Li et al. [67], except that lutein measurement was carried out using ultra (u)HPLC analysis. A value of about 7.7 mg/g dry weight, equal to 38.5% (*w*/*w*) astaxanthin content was found, in agreement with Vidhyavathi et al. [68], who found that, for the HPR cystic phase, lutein was about 30–40% (*w*/*w*) astaxanthin content. HPR was stored at −20 °C in a vacuum-sealed plastic bag to avoid degradation, and was brought to atmospheric conditions before extraction. All chemicals and standards (astaxanthin and lutein) were of analytical grade and purchased from Sigma Aldrich (St. Louis, MO, USA). All other reagents were of uHPLC grade unless otherwise stated. Carbon dioxide (industrial grade) with a purity of 99.999% was purchased from Rivoira (Bari, Italy).

### 3.2. Experimental Set-Up 

**Figure 5:**
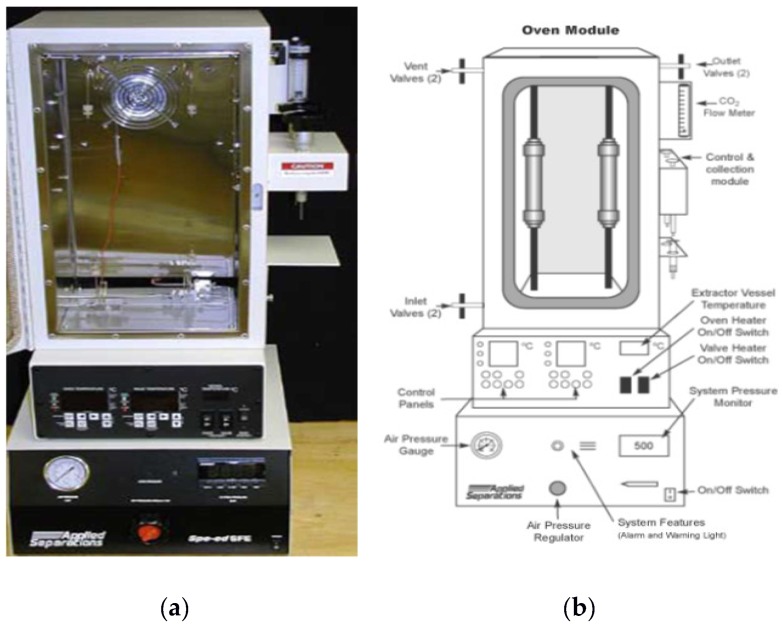
(**a**) Picture of the CO_2_ superfluid extractor (CO_2_-SFE) 2; (**b**) schema of bench-scale CO_2_-SFE.

The experimental set-up, shown in Figure 5, was a laboratory-scale equipment with a reactor volume of 30 mL [64]. The bench-scale experimental apparatus for CO_2_-SFE was equipped with a heater in order to achieve temperatures up to 250 °C, and a pumping system for the compression of CO_2_ up to 680 bar. Two vessels were located inside the module: the first was used as a CO_2_ pre-heater and the second was used for extraction. In the extraction vessel, two pressure control systems—inlet and outlet valves (Wika Transmitter, Klingenberg, Germany), with precision of 0.6 mbar—were installed, whereas the CO_2_ flow rate was controlled using a flow meter LPN/S80 AL G 2.5 (SACOFGAS, Milano, Italy). The inlet flow rate was adjustable up to 25 mL/min and the flow control was carried out on the expanded gas. Temperature was monitored using thermocouples, while the inlet and outlet flow streams were controlled using micrometric valves. The experimental apparatus was also equipped with a specific line for supplying co-solvent, using a syringe pump (Speed SFE Modifier Pump Module-PN 7170, Allentown, PA, USA) to compress the co-solvent up to 680 bar and to regulate the flow rate up to 10 mL/min. 

The experimental apparatus was also equipped with acoustic and visual high-pressure alerts and, as the primary security system, a rupture disc was installed. All parameters of the process were controlled using a distributed control system (DCS). Before each extraction stage, HPR was mechanically pre-treated using the Mixer Mill (Retsch MM400, Haan, Germany) with three steel spheres of 1 cm for 5 min at 400 rpm, in which 1.4 g of HPR was mixed with 0.8 g of diatom land [69]. 

For each experimental test, four extraction cycles of 20 min each (extraction time = 20–80 min) were carried out. Carbon dioxide and ethanol flow rates were kept constant at 3.62 g/min and 1 mL/min, respectively. Temperature (*T*) and pressure (*P*) were varied in the ranges of 50–80 °C and 100–550 bar, respectively. The experimental plan, in which HPR biomass loading was included, is reported in the Table 7.

The effects of pressure and temperature on astaxanthin and lutein extraction were reported in terms of cumulative recovery and purity [57]. Cumulative recovery was expressed both as mg of the compound extracted for g of dry HRP biomass loading (Equation (1)) and as percentage of the total content (Equation (2)). Purity was calculated as the ratio between the recovery (mg/g) and the total extraction yield (Equation (3)).

(1) $$\mathrm{Cumulative}\;\mathrm{recovery}\;\left(\%\right)=\frac{\left(\sum_{i}W_{c,i}\right)}{W_{B}}$$

(2) $$\mathrm{Cumulative}\;\mathrm{recovery}\;\left(\%\right)=\left\{\left[\frac{\left(\sum_{i}W_{c,i}\right)}{W_{B}}\right]/W_{T}\right\}\times 100$$

(3) $$\mathrm{Purity}\;\left(\%\right)=\left\{\left[\frac{\left(\sum_{i}W_{c,i}\right)}{W_{B}}\right]/W_{E}\right\}\times 100$$

where *W*_C,i_ (mg) is the weight of the compound extracted for each extraction cycle, *W*_B_ (g) is the weight of HPR biomass loading (Table 7), *W*_T_ (mg/g) is the total content of the compound (Section 3.1; 20.0 mg/g dry weight for astaxanthin; 7.7 mg/g dry weight for lutein), and *W*_E_ (mg/g) is the total extraction yield (Table 1).

Each experimental condition was investigated in triplicate. 

### 3.3. Analytical Methods

After each extraction cycle, the sample was collected in an amber vial and subjected to basic hydrolysis in the presence of NaOH (saponification), in order to remove lipids and chlorophylls from the sample, avoiding the overlap of the spectra with the species present in the carotenoid family [56]. Specifically, saponification was carried out by adding 1 mL of NaOH solution in methanol (0.05 M) to 5 mL of extract. This solution was left in the dark in an inert atmosphere for 7 h. Once this step was completed, the sample was neutralized with 3 mL of an NH_4_Cl solution in methanol (0.05 M). After saponification, astaxanthin and lutein were measured using a uHPLC Agilent 1290 Infinity II with a Zorbax reverse-phase C18 column with methanol/water (95:5, *v*/*v*) as a mobile phase solvent, while the sample was dissolved in a mixture of methanol/chloroform (90:10 containing 0.1% butylated hydroxytoluene (BHT) as an antioxidant). The flow rate and column temperature were kept constant at 0.4 mL/min and 28 °C, respectively [69]. The extraction procedure was detailed previously in Di Sanzo et al. [57].

## 4. Conclusions

In the present work, the effects of time (20–80 min), temperature (50–80 °C), and pressure (100–550 bar) on astaxanthin and lutein extraction from *H. pluvialis* in the red phase via CO_2_-SFE with ethanol (GRAS solvent) as a co-solvent were investigated. Moreover, a comparison of CO_2_-SFE with without ethanol was also carried out. 

The results highlight that, for both astaxanthin and lutein, temperature played a crucial role in both extraction yield and carotenoid degradation. At 65 °C and 550 bar, astaxanthin and lutein extraction yields were maximized, avoiding carotenoid thermal degradation. Moreover, at the best extraction conditions (65 °C and 550 bar), a negligible effect of extraction time was observed; therefore, to basically achieve the maximum recovery, an extraction time of 20 min was sufficient for astaxanthin, while an extraction time of 40 min was required for lutein. These conditions led to an astaxanthin extraction yield of about 92%, and a lutein extraction yield of about 93%. From the comparison between experimental findings and literature data, it is possible to see that the operative conditions identified in our study, including the type of pre-treatment, produced the highest recovery of astaxanthin and lutein. In terms of purity of astaxanthin, similar results were achieved.

By comparing the performance of CO_2_-SFE with and without ethanol, it is worth highlighting that a real effectiveness of ethanol as a co-solvent for astaxanthin extraction was not observed, with the drawback of a reduction in astaxanthin purity. On the other hand, the presence of ethanol was found to be effective for the extraction of lutein, as an enhancement was observed both in terms of recovery and purity.

## Figures and Tables

**Table 1:** Extraction yield during CO_2_ superfluid extraction (CO_2_-SFE) with and without co-solvent [57].

Operative Conditions		Total Extraction Yield (mg/g) (Ethanol Flow Rate = 1 mL/min)	Total Extraction Yield (mg/g) (Without Co-Solvent)
Extraction Time = 80 min			
CO_2_ Flow Rate = 3.62 g/min			
*T* (°C)	*P* (bar)		
50	100	209.99	0.1
50	400	235.47	132.4
50	550	241.64	234.8
65	100	241.62	4.8
65	400	292.70	277.1
65	550	280.78	184.6
80	100	207.67	10.6
80	400	253.03	158.7
80	550	222.57	59.4

**Table 2:** Comparison between astaxanthin cumulative recovery with and without co-solvent [57].

CO_2_ Flow Rate = 3.62 g/min; Extraction Time = 80 min						
*T* (°C)	*P* (bar)					
	100	400	550	100	400	550
	Cumulative Recovery with Co-Solvent (%) (Ethanol flow rate = 1 mL/min)			Cumulative Recovery without Co-Solvent (%)		
50	24.5	27.8	27.9	<0.01	91.6	94.5
65	38.3	74.2	92.4	0.05	79.8	35.2
80	8.4	10.1	14.5	5.7	72.7	13.5

**Table 3:** Purity of astaxanthin for each stage under different operative conditions with and without co-solvent [57].

CO_2_ Flow Rate = 3.62 g/min								
*T* and *P*	Astaxanthin Purity (%) with Co-Solvent				Astaxanthin Purity (%) without Co-Solvent			
	20 min	40 min	60 min	80 min	20 min	40 min	60 min	80 min
50 °C 100 bar	0.86	10.33	5.13	4.85	0	0	0	0
50 °C 400 bar	2.71	1.34	1.55	1.84	11.81	29.41	32.96	32.48
50 °C 550 bar	2.33	1.21	4.82	8.60	7.30	17.31	22.57	26.67
65 °C 100 bar	3.28	2.01	0.53	1.18	0	0	0	0
65 °C 400 bar	5.24	4.39	0.01	0.01	4.96	20.86	28.53	29.46
65 °C 550 bar	6.67	0.00	0.40	0.08	2.42	15.21	17.75	27.14
80 °C 100 bar	0.18	0.89	2.86	1.96	0	0	0	0
80 °C 400 bar	0.72	18.11	0.06	0.08	7.70	13.32	29.11	34.23
80 °C 550 bar	1.02	14.03	0.03	0.19	2.21	8.03	19.63	22.15

**Table 4:** Comparison between lutein cumulative recovery with and without co-solvent [57].

CO_2_ Flow Rate = 3.62 g/min; Extraction Time = 80 min						
*T* (°C)	*P* (bar)					
	100	400	550	100	400	550
	Cumulative Recovery with Co-Solvent (%) (Ethanol Flow Rate = 1 mL/min)			Cumulative Recovery without Co-Solvent (%)		
50	50.5	53.7	57.5	0.1	44.5	50.9
65	50.4	86.6	92.9	0	33.9	37.7
80	13.2	18.2	32.8	1.6	17.3	1.2

**Table 5:** Purity of lutein for each stage under different operative conditions with and without co-solvent [57].

CO_2_ Flow Rate = 3.62 g/min								
*T* and *P*	Lutein Purity (%) with Co-Solvent				Lutein Purity (%) without Co-Solvent			
	20 min	40 min	60 min	80 min	20 min	40 min	60 min	80 min
50 °C 100 bar	0.61	2.88	1.94	1.90	0	0	0	0
50 °C 400 bar	1.44	0.93	1.04	1.09	2.27	3.89	6.36	7.54
50 °C 550 bar	1.18	5.93	3.51	0.33	1.58	2.83	2.04	4.63
65 °C 100 bar	1.08	0.98	0.29	0.44	0	0	0	0
65 °C 400 bar	1.36	6.78	0.06	0.03	0.93	0.96	1.52	3.62
65 °C 550 bar	1.73	16.57	0.35	0.28	1.64	0.68	2.31	2.80
80 °C 100 bar	0.33	0.58	0.50	0.43	2.33	0.92	0.63	0.67
80 °C 400 bar	0.39	5.17	0.16	0.28	0.83	0.59	1.25	1.44
80 °C 550 bar	0.76	4.89	0.08	0.08	0	0.32	1.62	1.66

**Table 6:** Comparison of operative conditions for astaxanthin and lutein recovery from *Haematococcus pluvialis*.

Optimum Extraction Conditions							Carotenoid Recovery ^e^	Reference
Biomass Loading (g)	CO_2_ Flow Rate (g/min)	Co-Solvent ^a^	Pre-Treatment	*P*^b^ (bar)	*T*^c^ (°C)	*t*^d^ (h)		
n.a. ^§^	100 µL·min^−1^	20% (*v*/*v*) ethanol	Hydrotermal	80	55	15 min	Astaxanthin 98.3%	[50]
n.a. ^§^	100 µL·min^−1^	20% (*v*/*v*) olive oil	Hydrotermal	80	55	15 min	Astaxanthin 98.6%	
7	1.41 g/min	5% (*v*/*v*) ethanol	Drying	400	70	4	Astaxanthin 77.9%	[53]
2	1.41 g/min	10% (*v*/*v*) ethanol	Freeze drying and ball milling	300	60	-	Astaxanthin >90%; Lutein >90%	[52]
6	1.41 g/min	10% (*v*/*v*) olive oil	Drying	400	70	5	Asthaxanthin 36%	[61]
240	7.8 g/min	2.3 mL/g sample ethanol	Freeze drying (powder form)	435	65	3.5	Astaxanthin 87.42%	[66]
1.38	3.62 g/min	12.5% (*v*/*v*) ethanol	Ball milling	550	65	1.33 (20 min for Astaxanthin; 40 min for Lutein) ^#^	Astaxanthin 92.4%; Lutein 92.9%	This study

^a^ Ethanol/vegetable oils mentioned in the column served as a co-solvent in the extraction; ^b^ operating pressure; ^c^ operating temperature; ^d^ total extraction time; ^e^ recovery at optimum conditions; ^§^ n.a. = not available; ^#^ at the operative conditions with which the maximum recoveries were achieved (65 °C; 550 bar), the recoveries were not affected by extraction time.

**Table 7:** CO_2_-SFE extraction conditions. HPR—*H. pluvialis* in the red phase.

Operative Conditions		
*T* (°C)	*P* (bar)	HPR Biomass Loading (g)
50	100	1.43
50	400	1.43
50	550	1.37
65	100	1.36
65	400	1.36
65	550	1.38
80	100	1.35
80	400	1.38
80	550	1.34
